# Investigation of the effects of the magnetic field on the anodic dissolution of alloy 690 in SO_4_^2−^ + SCN^−^ solution using digital holography

**DOI:** 10.1016/j.heliyon.2023.e13566

**Published:** 2023-02-09

**Authors:** Dongling Xu, Chen Sang, Boyu Yuan, Liang Li

**Affiliations:** a Jiangsu Key Laboratory of Green Synthetic Chemistry for Functional Materials, School of Chemistry & Materials Science, Jiangsu Normal University, Xuzhou, 221116, China; b Jiangsu Key Laboratory of Advanced Laser Materials and Devices, School of Physics and Electronic Engineering, Jiangsu Normal University, Xuzhou, 221116, China

**Keywords:** Alloy 690, Magnetic field, Digital holography, Anodic dissolution, SO_4_^2−^ + SCN^−^

## Abstract

Digital holography has been employed for *in situ* observation of dynamic processes occurring at the electrode|electrolyte interface during the anodic dissolution of Alloy 690 in solutions containing SO_4_^2−^ + SCN^−^ with or without magnetic field (MF). It was found that MF increased the anodic current of Alloy 690 in 0.5 M Na_2_SO_4_ + 5 mM KSCN solution but showed a decreased value when evaluated in 0.5 M H_2_SO_4_ + 5 mM KSCN solution. For each solution, as a result of the stirring effect due to Lorentz force, MF showed a decreased localized damage further preventing pitting corrosion. The content of nickel and iron at grain boundaries is higher than that on the grain body, in accordance with the Cr-depletion theory. MF increased the anodic dissolution of nickel and iron, which in turn increased the anodic dissolution at grain boundaries. *In situ* inline digital holography revealed that IGC begins at one grain boundary and progresses to adjacent grain boundaries with or without MF.

## 1 Introduction

High-chromium nickel alloy i.e. Alloy 690 (wt%, Cr-29.1, Fe-9.0, Ni-balance) was created for use in industries like the chemical industry, food processing, nuclear power plants, and is frequently used for steam generator tubing applications in pressurized water reactors (PWRs) due to its high corrosion resistance and superior mechanical strength [1]. Alloy 690 is prone to pitting and intergranular corrosion (IGC) in the presence of the aggressive ions including Cl^−^ and SCN^−^ even though it exhibits excellent corrosion resistance in most conditions. IGC can weaken an alloy and increase the risk of accidents [2].

Researchers studied the effects of SO_4_^2−^ on the anodic dissolution of the Fe–Ni alloy for its importance in nuclear power plants as a coolant. Zuo et al. [3] used potentiostatic and potentiodynamic polarization approaches to study the effect of SO_4_^2−^ ions on metastable pitting corrosion of 316L stainless steel. They discovered that SO_4_^2−^ slowed the metastable pitting's nucleation and propagation rates. Yang [4] investigated the anodic dissolution of Alloy 690 at 316 °C in solutions containing sulfate and chloride ions, and he discovered that 8000 ppm sulfate ions inhibited the alloy's pitting corrosion in the presence of 8000 ppm chloride ions at pH 4. Maximovitch et al. [5] studied the electrochemical behaviour of nickel-based alloys in neutral and slightly alkaline sulfate solutions at 320 °C and found that chromium oxide exhibits tremendous corrosion resistance. Luo et al. [6] investigated the passive film structure formed on Inconel 800 in sulfate and chlorine solutions and proposed that their semiconductor properties were largely dependent on the competitive adsorption of SO_4_^2−^ and Cl^−^ ions. Evans and Hart [7] thoroughly studied the corrosion and passivation of a Ni–Si-based alloy in boiling H_2_SO_4_ solution by using electrochemical methods, and they proposed that the alloy exhibits a low rate of corrosion at all concentrations except within a narrow range between 47 and 55 wt%. Badawy et al. [8] explored the stability of Cu–Al–Ni alloys in Na_2_SO_4_ + NaCl solution at 25 °C, and they discovered that when the Ni-level in the ternary alloy increased, a stable passive film began to emerge. However, the stability of the protective film was affected by the Cl^−^ ions in the media. Using the single-loop electrochemical potentio-kinetic reactivation (SL-EPR) approach, Wu et al. [9] discussed the reactivation behaviour of Alloy 600 in H_2_SO_4_+KSCN solution and discovered a maximum current peak associated with IGC at a concentration ratio of 10 for H_2_SO_4_/KSCN. Wang et al. [10] examined the grain-oriented dissolution of polycrystalline Alloy 690 in H_2_SO_4_ + NaCl solution, as demonstrated by scanning electron microscopy (SEM) and atomic force microscopy (AFM). Researchers noticed that the alloy tended to dissolve in the directions with lower surface energy.

SCN^−^ ions (sensitizer) are used for measuring the IGC resistance of the metallic materials. Casales et al. [11] investigated the IGC resistance of Alloy 690 in 0.5 M H_2_SO_4_ + 0.0001 M KSCN solution using the dual-loop electrochemical potentio-kinetic reactivation (DL-EPR) technique; in the investigation, the greatest corrosion was observed in the chromium-depleted zone near grain boundaries. Tsai et al. [12] investigated the reactivation behaviour of Inconel 600 in H_2_SO_4_ + KSCN solution by the SL-EPR method in which a maximum anodic current associated with IGC was observed when the H_2_SO_4_/KSCN concentration ratio was 10.

Magnetoelectrochemistry has been studied for several decades due to its potential applications in industry. Three forces, including the paramagnetic gradient force (**F** ∇_**C**_), the Lorentz force (**F**_**L**_), and the gradient magnetic force (**F** ∇_**B**_), are applied under MF in an electrochemical reaction [13,14]. **F**_**L**_ is much higher than **F** ∇_**B**_ when the electrode surface is parallel to uniform MF [15], which enhances the mass transport in the electrochemical reaction, as **F** ∇_**C**_ is neglected at room temperature [16].

The aforementioned research primarily employed traditional techniques, such as voltammetry, electrochemical impedance spectroscopy, and *ex situ* physical surface approaches, including XPS and SEM. Traditional methods are used to demonstrate the surface morphology, corrosion products, and overall behaviour of an electrochemical system; nevertheless, it is challenging to examine the specifics of how local corrosion works. *In situ* methods are well known strategies to study the corrosion mechanism of metallic materials. Recently, in-line digital holography (*in situ* approach) was used to study the anodic dissolution of the Ni|H_3_PO_4_ + SCN^−^ system with and without MF [17], further showing an enhanced IGC under MF. The anodic dissolution of Alloy 690 in neutral and alkaline NaCl solutions with or without MF was examined using in-line digital holography. It was shown that MF increased IGC even though it decreased the anodic current [18]. In-line digital holography can be used to *in situ* observe the dynamic processes at the interface during an electrochemical reaction and explain the effects of MF on the anodic dissolution of metallic materials [15,[17], [18], [19], [20]].

Due to the occurrence of IGC by SCN^−^ in Ni-based alloys and usage of seawater containing SO_4_^2−^ as a coolant in nuclear power plants, the IGC of Alloy 690 was studied in SO_4_^2−^ + SCN^−^ solution. To our knowledge, few studies have been performed on the anodic dissolution of Alloy 690 in neutral SO_4_^2−^ + SCN^−^ solution by using *in situ* techniques. In this work, modern in-line digital holography was used in combination with the conventional electrochemical method to observe the dynamic processes at the electrode-electrolyte interface during the anodic dissolution of Alloy 690 in neutral SO_4_^2−^ + SCN^−^ solution *in situ*. The MF effects on the anodic dissolution of Alloy 690 in neutral and acidic SO_4_^2−^ + SCN^−^ solutions were discussed in detail.

## 2 Methods

### 2.1 Electrochemical system

The working electrodes for the three-electrode system (as shown in Fig. S1) were prepared with an Alloy 690 wire (d = 1 mm, with the composition of Cr-29.1%, Fe-9.0%, Nb-0.96%, Mn-0.79%, Mo-0.42%, Si-0.16%, Ni-balance, Shenzhen AVIC Special Alloy Co., Ltd. P. R. China) sealed with epoxy resin in a glass tube. The cross-section of the wire was exposed to the solution as the surface of the working electrode. The counter and reference electrodes were a platinum sheet (10 mm × 10 mm) and saturated calomel electrode (SCE), respectively. A Lugging capillary was placed between the working and reference electrodes for IR drop reduction.

The electrochemical analysis was carried out by a CHI 660E electrochemical workstation (CH Instruments, Inc., Austin, TX, USA), and the surface morphologies were observed using an SEM (Hitachi S–3400 N SEM, Tokyo, Japan) equipped with EDS. The current study was executed at room temperature (25 °C ± 2 °C).

The working electrode was gradually abraded with 600#, 1200#, and 2000# metallographic sandpapers and cleaned in an ultrasonic bath with distilled water followed by ethanol. The solutions were prepared from distilled water and analytical-grade reagents. Two permanent magnets (200 mT) were used in the experiments.

### 2.2 Recording system

The holographic recording system was the same as that used in our previous study [19], as shown in Fig. S2. The soluble corrosion products changed their concentration (Δc) at the interface during anodic dissolution, followed by a corresponding change in refractive index (Δn) and phase difference (ΔΦ). ΔΦ, Δc, and Δn are related by Equation (1) [20].

(1) Δc = kΔn = kλ_0_ΔΦ/(2πd)

where k, λ_0_, and d are the concentrative refractivity, light wavelength, and geometrical path length, respectively.

## 3 Results

### 3.1 Tafel plots

#### 3.1.1 Alloy 690 in 0.5 M Na_2_SO_4_ + 0.005 M KSCN solution (neutral)

**Fig. 1:**
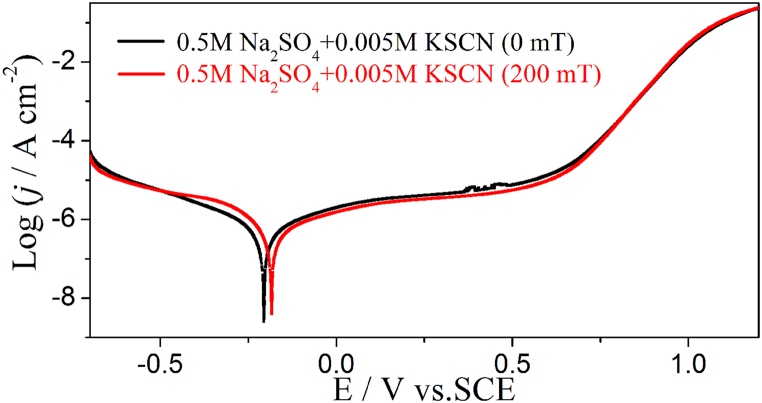
The Tafel curves of Inconel 690 in 0.5 M Na_2_SO_4_ + 0.005 KSCN solutions without and with MF, respectively, scan rate: 1 mV/s.

Fig. 1 shows the Tafel curve of Alloy 690 in 0.5 M Na_2_SO_4_ + 0.005 M KSCN solution (neutral) with and without a MF. The MF increases the cathodic current when E > −0.5 V in the cathodic branch and decreases the anodic current when E < 0.8 V, but little effect is observed when E > 0.8 V in the anodic branch. The parameters corresponding to Fig. 1 are listed in Table 1. The corrosion parameters are obtained by cathodic Tafel fitting only because the anodic Tafel characteristics were not obvious. The results show that the positive corrosion potential (E_corr_) and pitting potential (E_pit_) shift under MF because of an increase in cathodic current and a decrease in anodic current.

**Table 1:** The relevant parameters of the Tafel curves in 0.5 M N_2_SO_4_ + 0.005 M KSCN (Fig. 1).

B/mT	E_corr_/V	j_corr_/μA cm^−2^	−b_c_/mV	E_pit_/V
**0**	−0.205	0.847	112.4	0.519
**200**	−0.182	1.71	92.4	0.548

#### 3.1.2 Alloy 690 in 0.5 M H_2_SO_4_ + 0.005 M KSCN solution (acidic)

**Fig. 2:**
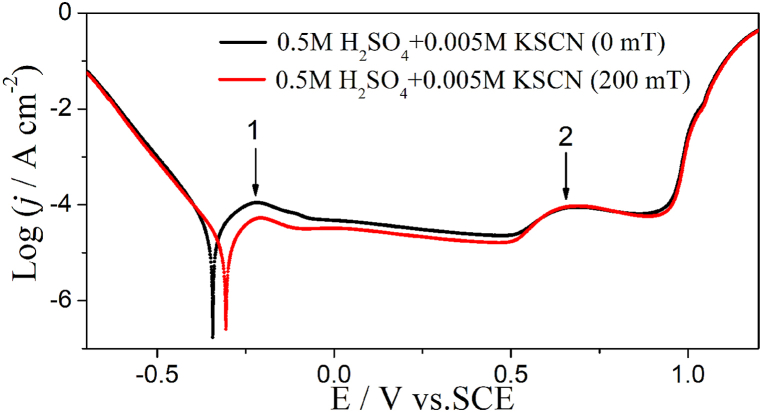
The Tafel curves of Inconel 690 in 0.5 M H_2_SO_4_ + 0.005 KSCN solutions without and with MF, respectively, scan rate: 1 mV/s.

Fig. 2 shows the Tafel curve of Alloy 690 in 0.5 M H_2_SO_4_ + 0.005 M KSCN solution (acidic) with and without MF. MF hardly affects the cathodic current when E > −0.5 V in the cathodic branch. In the anodic branch, the first current peak (j_p1_) appears at −0.20 V (E_p1_) and MF decrease the current till the second current peak (j_p2_) appears at 0.69 V (E_p2_). Table 2 lists the corresponding parameters from the Tafel curve (Fig. 2). MF causes a positive shift in E_corr_ and decreases j_corr_ because it has little effect on the cathodic reaction but inhibits the anodic reaction. MF affects E_pit_, E_p1_, and E_p2_ minutely in the acidic solution, and decreases j_p1_ drastically but increases j_p2_ slightly. As shown in Table 1, Table 2, a difference in the polarization curves depends on the pH of the solution.

**Table 2:** The relevant parameters of the Tafel curves shown in 0.5 M H_2_SO_4_ + 0.005 M KSCN (Fig. 2).

B/mT	E_corr_/V	j_corr_/μA cm^−2^	−b_c_/mV	E_pit_/V	E_p1_/V	j_p1_/μA cm^−2^	E_p2_/V	j_p2_/μA cm^−2^
**0**	0.342	44.1	95.3	0.503	−0.215	108	0.69	89.5
**200**	0.306	18.2	83.1	0.505	−0.207	53.7	0.693	94.2

### 3.2 Polarization curves and the corresponding phase maps

#### 3.2.1 Alloy 690 in 0.5 M Na_2_SO_4_ + 0.005 M KSCN solution (neutral)

**Fig. 3:**
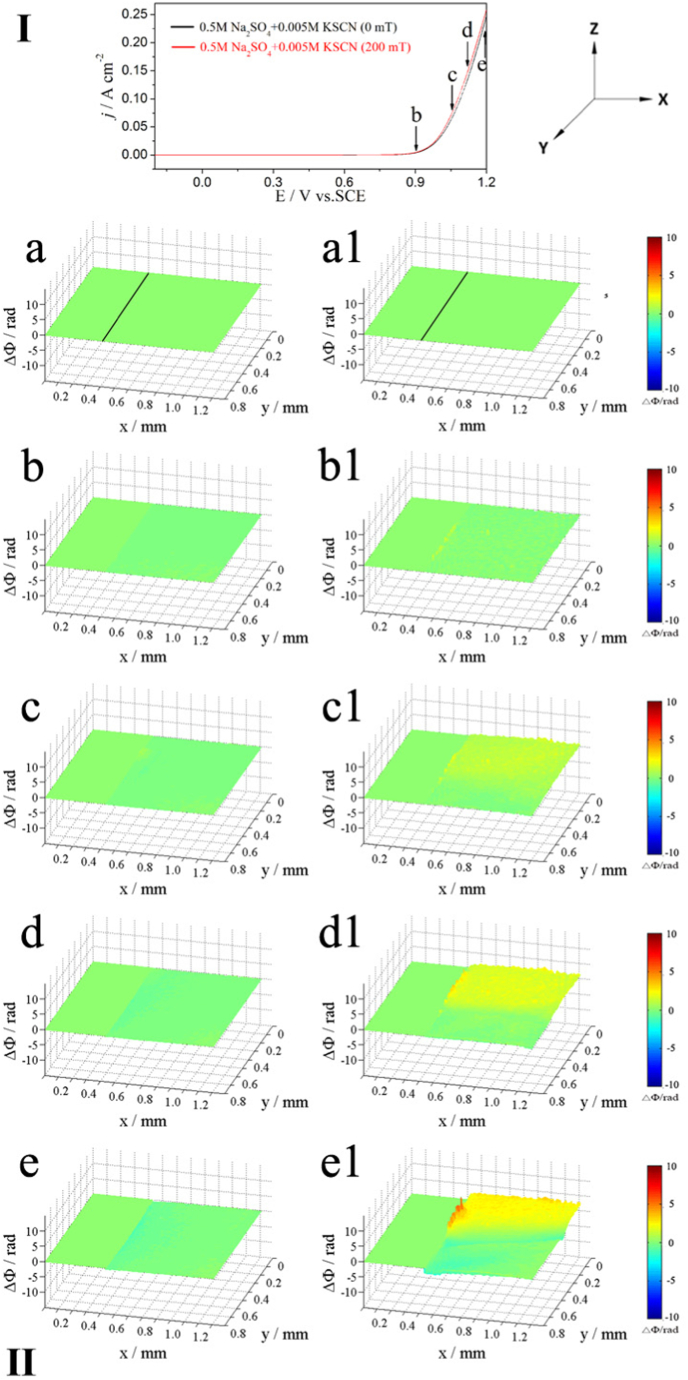
The polarization curves (upper part, Ⅰ) of Inconel 690 in 0.5 M Na_2_SO_4_ + 0.005 KSCN solutions without and with MF and the phase maps (lower part, Ⅱ) at different potentials corresponding to points b-e in the polarization curves, a and a1 obtained at the open circuit potential (the same below).

Fig. 3 shows the potentiodynamic polarization curves (I) and corresponding phase maps (II) of Alloy 690 in 0.5 M Na_2_SO_4_ + 0.005 M KSCN solution with and without MF at a scanning rate of 10 mV/s. Thus, each axis represents a specific direction. The horizontal, optical and gravity opposite directions are the individual representations for the x-, y- and z-axes. The polarization curve (I) (Fig. 3) shows a narrow influence of the MF on the anodic current (when E < 0.9 V) due to the low Lorentz force. However, the anodic current increases with a positive potential shift when E > 0.9 V under MF and promotes anodic dissolution.

Fig. 3a and a1 display the phase map with a black line between the electrode (left) and the solution (right), obtained at the open circuit potential (OCP). The higher the ΔΦ, the higher the concentration gradient (Δc). The relationship between Δc, ΔΦ, and the colours in the phase maps are listed in Table 3.

**Table 3:** Relationship between color, the phase difference, and concentration gradient corresponding to phase maps (Fig. 3, Fig. 4, Fig. 5, Fig. 6).

Color	Phase difference (ΔΦ)	Concentration Gradient (ΔC)
Blue	ΔΦ < 0	ΔC < 0
Green	ΔΦ ∼ 0	ΔC ∼ 0
Yellow/Red	ΔΦ > 0	ΔC > 0

Without the presence of MF, anodic dissolution began, as shown in phase maps (II) in Fig. 3b, in which the emergence of yellow light at the contact denotes the commencement of localized corrosion. As soon as the potential shifts to point c, the corrosion at the interface increases, and the interface shows two layers, inner yellow and outer blue (Fig. 3c). Upon reaching points d and e, only the blue layer appears at the interface that deepens with the increase in potential (Fig. 3d and e). With the presence of MF, localized corrosion is also observed (Fig. 3b1) at the beginning of dissolution. As the potential shifts to point c, the MF produces convection, making the concentration more uniform, and only yellow areas can be observed (Fig. 3c1). At points d and e, only yellow areas are observed (Fig. 3d1), and the MF induces strong convection at the interface (Fig. 3d1 and 3e1). MF induces convection in the blue areas when the potential is sufficient (Fig. 3e1).

#### 3.2.2 Alloy 690 in 0.5 M H_2_SO_4_ + 0.005 M KSCN solution (acidic)

**Fig. 4:**
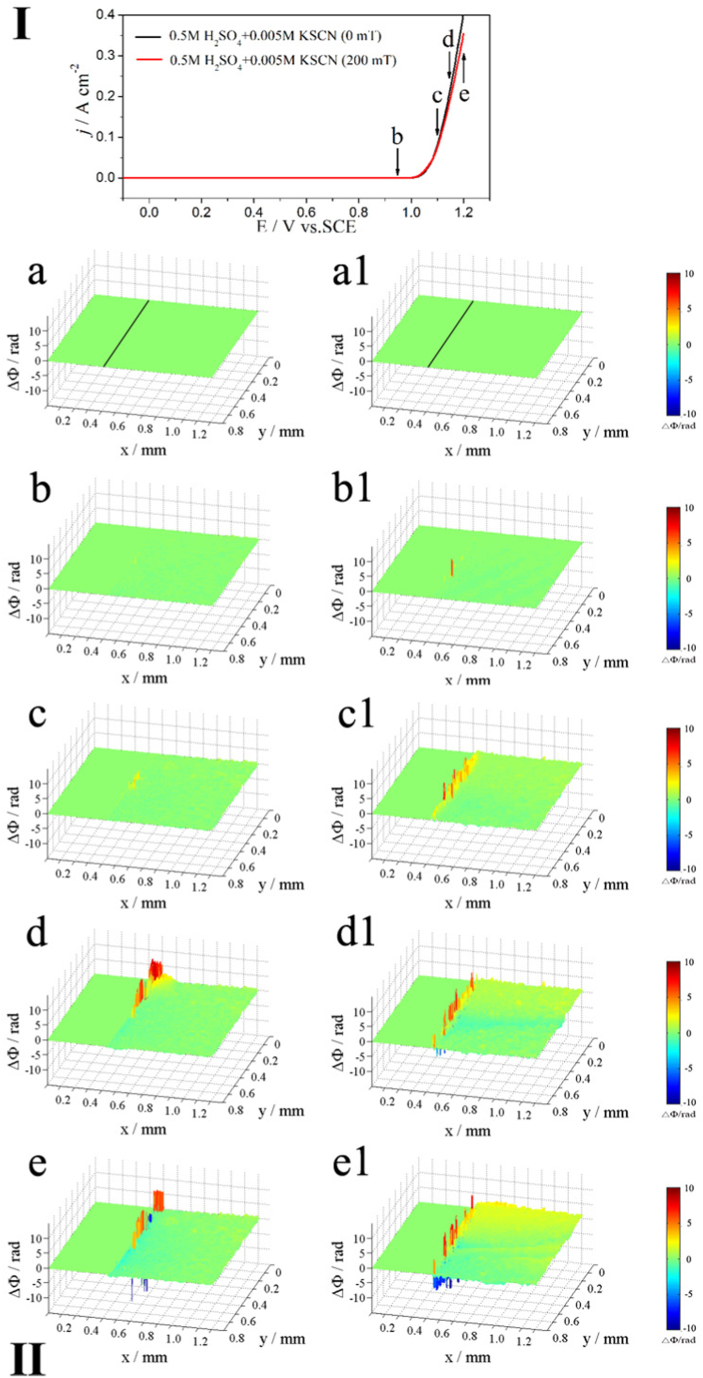
The polarization curves (upper part, Ⅰ) of Inconel 690 in 0.5 M H_2_SO_4_ + 0.005 KSCN solutions without and with MF and the phase maps (lower part, Ⅱ) at different potentials corresponding to points b-e in the polarization curves.

Fig. 4 shows the potentiodynamic polarization curves (I) and corresponding phase maps (II) of Alloy 690 in 0.5 M H_2_SO_4_ + 0.005 M KSCN solution under MF. Fig. 4 shows a low anodic current when E < 1.0 V, and the current increases when E > 1.0 V, indicating that the MF tends to decrease the current, whereas it increases with increasing potential.

Fig. 4a and a1 are obtained at OCP. Phase maps (II) show localized dissolution at the interface with or without MF (Fig. 4b and b1). When the potential positively shifts to point c, the localized concentration increases at the interface without an MF (Fig. 4c), whereas a more uniform concentration is observed under an applied MF (Fig. 4c1). After reaching points d and e without an MF, the inner yellow-red layer and the outer blue layer are observed at the interface, demonstrating that the concentration increases within the inner layer and decreases within the outer layer (Fig. 4d and e). The positive potential shift under MF induces convection in yellow, red, and blue regions that intensify with increasing potential (Fig. 4d1 and e1).

### 3.3 Effects of MF on the j–t curves

#### 3.3.1 Alloy 690 in 0.5 M Na_2_SO_4_ + 0.005 M KSCN solution (neutral)

**Fig. 5:**
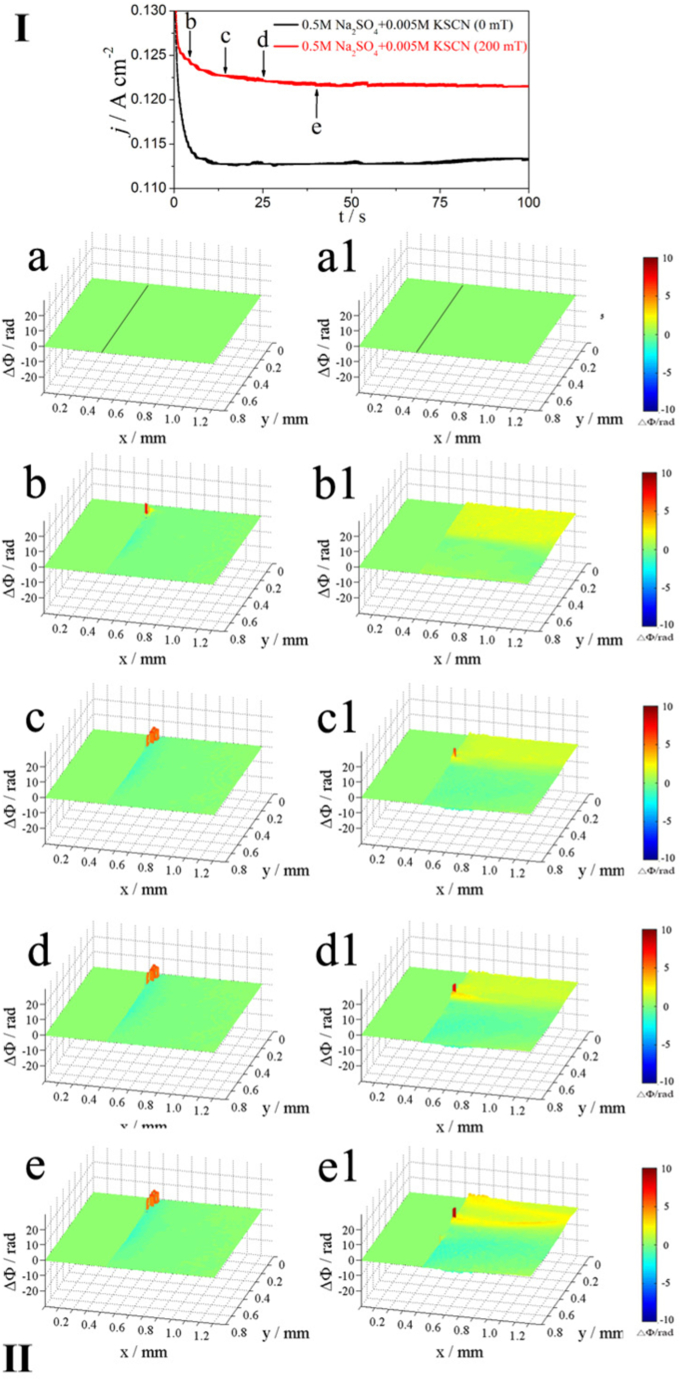
The *j-t* curves (upper part, Ⅰ) of Inconel 690 in 0.5 M Na_2_SO_4_ + 0.005 KSCN solutions without and with MF at 1.1 V and the phase maps (lower part, Ⅱ) at different times corresponding to points b-f in the *j-t* curves.

Fig. 5 shows the j-t curve (I) and corresponding phase maps (II) of Alloy 690 in 0.5 M Na_2_SO_4_ + 0.005 M KSCN solution at 1.1 V. Points b and c show how the current first declines before approaching a steady state, i.e., point e. Through a 9% increase in current, MF encourages anodic breakdown.

Fig. 5a and a1 are obtained at OCP. The phase maps (II) in Fig. 5(b–e) show two regions at the interface (blue and local red and blue layers) under no MF. The build-up of soluble materials at the interface causes the local concentration, as shown in the yellow and red regions, to increase over time. In contrast, the local concentration, as shown in the blue area, decreases over time due to the creation of complex ions or the hydrolysis of metal ions. The two different regions (yellow and blue) at the interface appear when MF is applied (Fig. 5b1–e1). Due to convection caused by MF, the blue region becomes brighter and growes over time, while the yellow and red portions continue to contract (Fig. 5b1–e1).

#### 3.3.2 Alloy 690 in 0.5 M H_2_SO_4_ + 0.005 M KSCN solution (acidic)

**Fig. 6:**
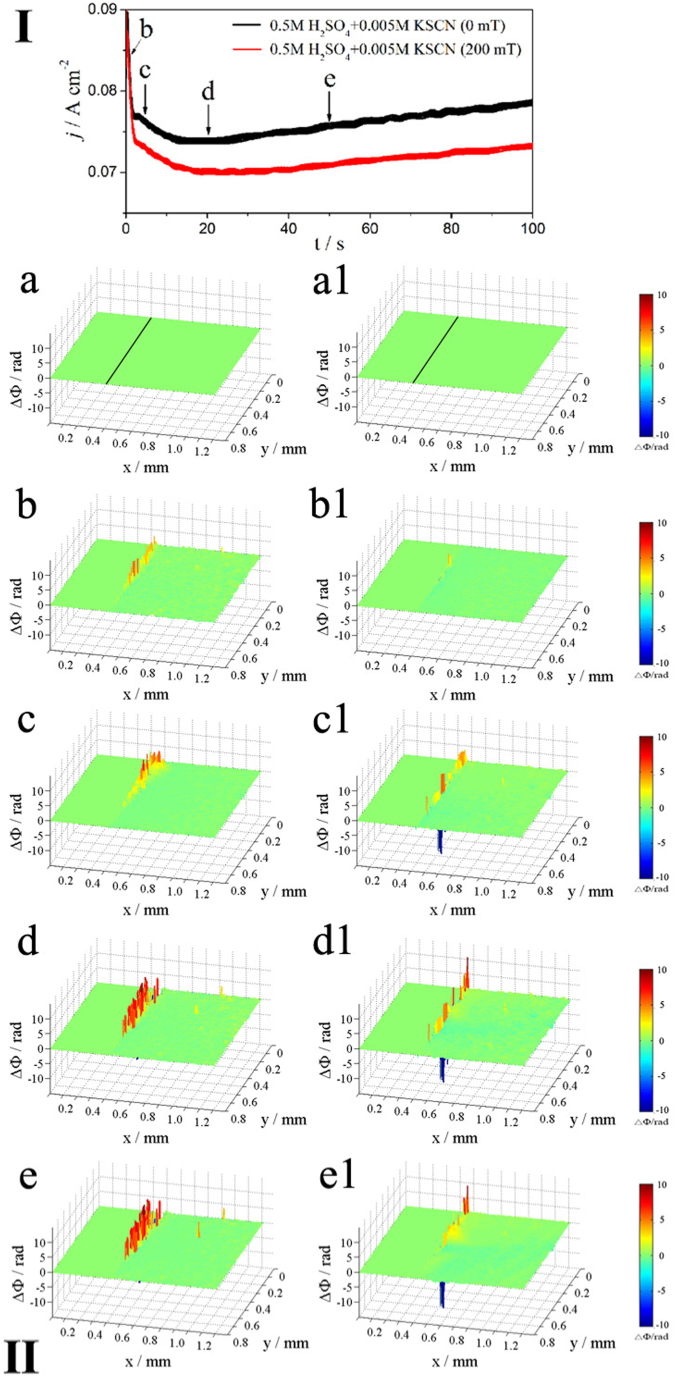
The *j-t* curves (upper part, Ⅰ) of Inconel 690 in 0.5 M H_2_SO_4_ + 0.005 KSCN solutions without and with MF at 1.1 V and the phase maps (lower part, Ⅱ) at different times corresponding to points b-f in the *j-t* curves, respectively.

Fig. 6 shows the j-t curve (I) and the corresponding phase maps (II) of Alloy 690 in 0.5 M H_2_SO_4_ + 0.005 M KSCN solution at E = 1.1 V with and without MF. The j-t curve (I) shows the reduction in current further inhibiting anodic dissolution under an applied MF. The anodic current is much lower in an acidic solution (Fig. 6) than in a neutral solution (Fig. 5).

Fig. 6a and a1 are obtained at OCP. The phase maps (II) in Fig. 6b–e shows the gradual increase in a local concentration at the interface, in which the absence of a blue area indicates an unchanged concentration. The local concentration increases at the interface under an MF (Fig. 6b1). The two layers (outer blue and inner yellow-red) are formed at the interface from point(s) c to e (Fig. 6c1–e1), in which the concentration gradually decreases in the blue areas with time. Under the MF, convection is produced in the yellow and red regions; however, compared to that in the neutral solution, convection is weaker in the acidic solution.

### 3.4 Surface analysis

#### 3.4.1 Alloy 690 in 0.5 M Na_2_SO_4_ + 0.005 M KSCN solution (neutral)

**Fig. 7:**
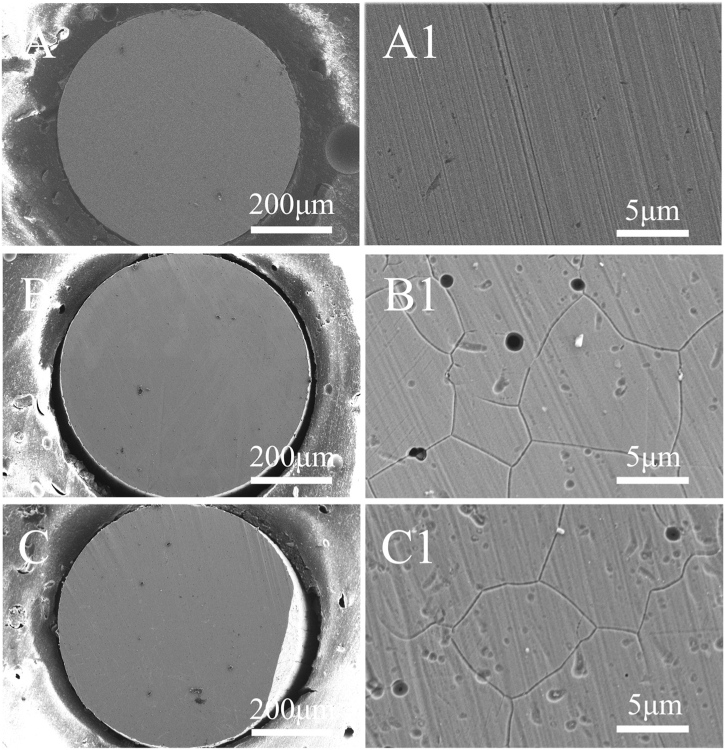
Surface morphologies of Inconel 690 recorded before (A and A1) and after potentiostatic polarization at 1.1 V for 50 s in 0.5 M Na_2_SO_4_ + 0.005 KSCN solutions without (B and B1) and with MF (C and C1).

Fig. 7 shows the surface morphologies of Alloy 690 before and after anodic dissolution at E = 1.1 V in 0.5 M Na_2_SO_4_ + 0.005 M KSCN solution with and without MF. Fig. 7A and A1 show the surface before the test. The surface shows scratches that were the result of grinding. The corrosion products are observed on the electrode surface after anodic dissolution without MF (Fig. 7B) and under MF (Fig. 7C) for 50 s. Fig. 7B1 and 7C1 show that the application of MF decreases the development of pits while enhancing the corrosion in grain boundaries, demonstrating the coexistence of IGC and pitting corrosion.

#### 3.4.2 Alloy 690 in 0.5 M H_2_SO_4_ + 0.005 M KSCN solution (acidic)

**Fig. 8:**
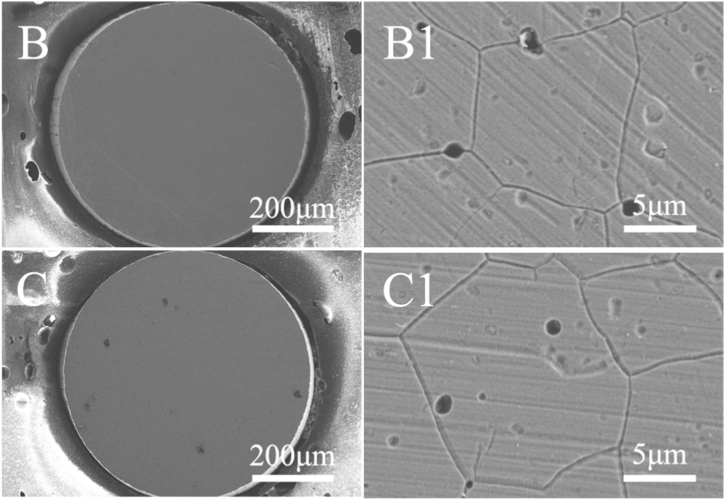
Surface morphologies of Inconel 690 after potentiostatic polarization at 1.1 V for 50 s in 0.5 M H_2_SO_4_ + 0.005 KSCN solutions without (B and B1) and with MF (C and C1).

Fig. 8 shows the surface morphologies of Alloy 690 after polarization at E = 1.1 V in 0.5 M H_2_SO_4_ + 0.005 M KSCN solution for 50 s with or without MF. Corrosion products (Fig. 8B and C) were formed on the surface of the electrode regardless of MF induction, in which IGC and pitting corrosion were observed. However, MF inhibits pitting corrosion but slightly promotes IGC.

### 3.5 EDS results

**Fig. 9:**
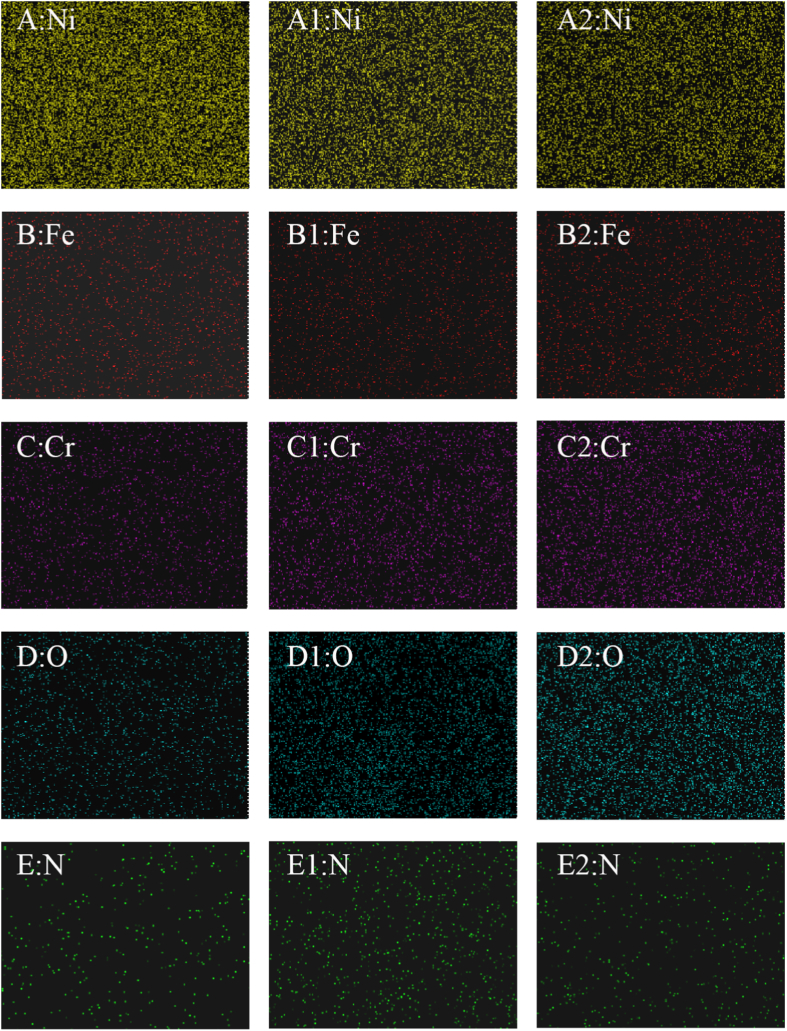
EDS results of the sample corresponding to Fig. 7 A1-C1. A–E: Blank; A1-E1: After anodic dissolution without MF; A2-E2: After anodic dissolution with MF.

**Fig. 10:**
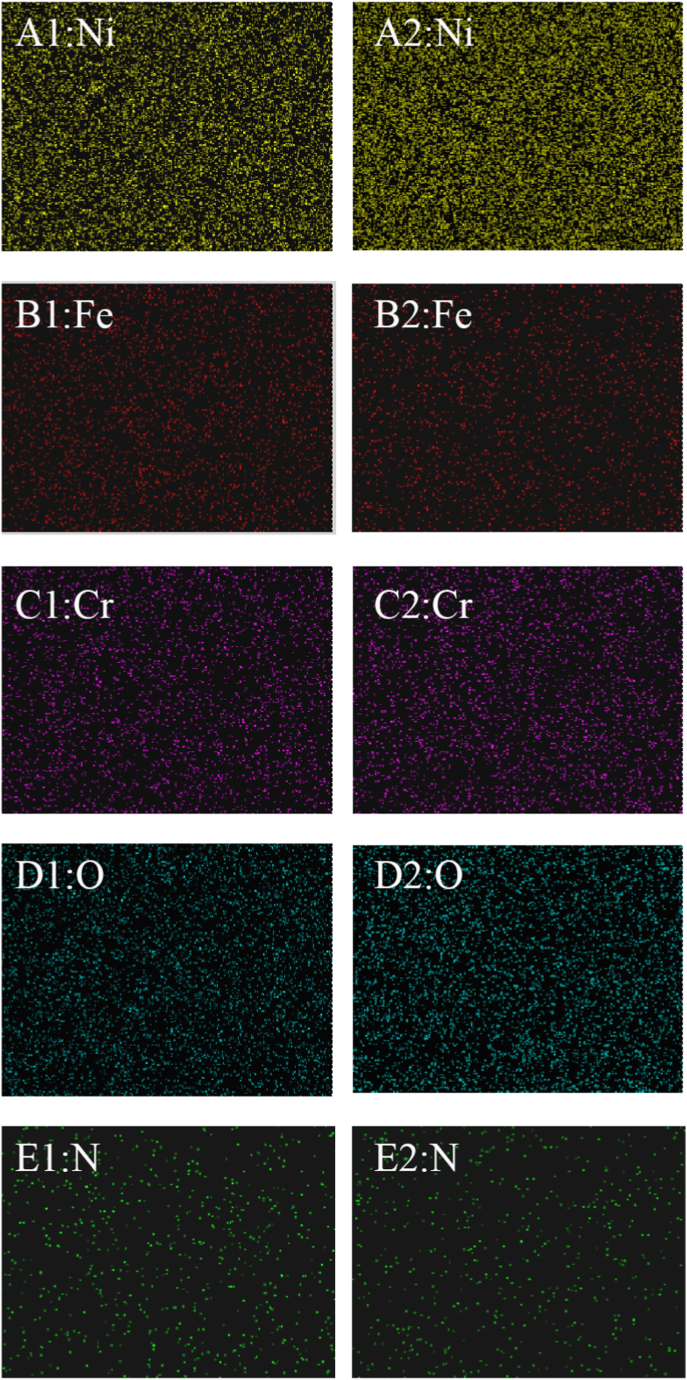
EDS results of the sample corresponding to Fig. 8 B1 and C1. A1-E1: After anodic dissolution without MF; A2-E2: After anodic dissolution with MF.

The chemical composition was measured by EDS. Fig. 9, S3, 10 and S4 demonstrate the EDS results of the sample corresponding to Fig. 7A1–C1 and Fig. 8B1, C1. Fig. 9 A–E, A1–E1, and A2–E2 show the content of Ni, Fe, Cr O, and N on the surface before the test, after anodic dissolution without MF and with MF, respectively. Fig. 10 A1–E1 and A2–E2 show the content of Ni, Fe, Cr O, and N on the surface after anodic dissolution without MF and with MF, respectively. As shown in Fig. 9, Fig. 10, in both the neutral and acidic solutions, the contents of oxygen and chromium are higher, indicating that the surface film is mainly composed of chromium oxide in both the neutral and acidic solutions. The following is a list of the effects of MF on the surface composition.

(1) The content of N is higher without an MF (Fig. 9 E1 and 10E1) than that with an MF (Fig. 9 E2 and 10E2) in each solution;
(2) The contents of O and Cr are slightly higher with an MF (Fig. 9C2 and D2, 10C2 and D2) but lower without an MF (Fig. 9C1 and D1, 10C1 and D1).
(3) An MF reduces the anodic current, and the content of Ni in acidic solutions is greater with an MF (Fig. 10 A2) compared to that without an MF (Fig. 10 A1); in neutral solutions, the amount of Ni with an MF is nearly identical to that without an MF.

## 4 Discussion

The anodic dissolution of Ni in a solution of chloride ions was reported and is illustrated in Equations (2), (3), (4) [16]:

(2) Ni + H_2_O = Ni(H_2_O)_ads_

(3) Ni(H_2_O)_ads_ + Cl^−^ = NiClH^+^ + OH^−^ + e^−^

(4) NiClH^+^ + Cl^−^ = NiCl_2_ + H^+^ + e^−^

The pseudohalogen SCN^−^ ion [21] is attributed mainly to the anodic dissolution of Ni in Alloy 690 (nickel-based alloy), as shown in Equations (5), (6), (7):

(5) Ni + H_2_O = Ni(H_2_O)_ads_

(6) Ni(H_2_O)_ads_ + SCN^−^ = NiSCNH^+^ + OH^−^ + e^−^

(7) NiSCNH^+^ + SCN^−^ = Ni(SCN)_2_ + H^+^ + e^−^

It remains unclear whether the Lorentz force (**F**_**L**_, Equation (8)) affects the electron transfer step. However, it is generally accepted that **F**_**L**_ promotes the mass transfer processes whether it increases or decreases the anodic current.

(8) **F**_**L**_ = j × **B**

where **B** and j are the MF intensity and the current, respectively.

As shown in Fig. 2, j_p1_ and j_p2_ may correspond to Equations (7) and (9). **F**_**L**_ may drive the active intermediate (NiSCNH^+^) away from the surface of the electrode [16] to inhibit the anodic dissolution of the alloy (reaction (6)) which is why F_L_ decreases j_p1_ (Table 2). Because F_L_ hardly affects the oxide film (Equation (9) [22]), it hardly affects j_p2_ (Table 2). Alloy 690 shows no passivation in either neutral or acidic SO_4_^2−^ + SCN^−^ solution in the current investigation (Fig. 1, Fig. 2). The current increases with the positive potential shift in the anodic branch when E > 1.0 V, and the rate-determining step is the electron transfer step at a high potential of 1.1 V.

(9) Ni(OH)_2_ + 2OH^−^ − 2e^−^ = NiO_2_ + 2H_2_O E^φ^ = 0.490 V vs. SHE

Alloy 690 produces Me(II) (Me = Ni and Fe) ions during anodic dissolution, which increases the concentration of the soluble species (corrosion products) at the interface (Fig. 3, Fig. 4, Fig. 5, Fig. 6), while the concentration decreases due to the formation of complexes between Me(II) and SCN^−^ ions. Fig. 3e1, 4e1, 5e1, and 6e1 show the convection that results from the stirring effect of the MF in blue regions. It was reported that [Ni(SCN)_4_]^2-^ [23] and [Fe(SCN)_4_]^-^ [24] complex ions were formed by SCN^−^ ions coordinating with Ni(Ⅱ) and Fe (Ⅲ) [25]. The concentration drops due to the creation of complex ions, and F_L_ produces convection of the electrolyte containing complex ions in the blue regions.

MF shows less anodic dissolution in the acidic solution compared to the other solutions for the following reasons.

(1) As shown in Fig. 10, oxide film (chromium oxide) formation on the electrode surface is promoted under an MF due to enhanced anodic dissolution of Ni and Fe with increasing Cr on the surface.
(2) Enhanced reaction (6) produces many NiSCNH ^+^ ions in the acidic solution. **F**_**L**_ may drive the active intermediate (NiSCNH^+^) away from the surface of the electrode [16] to inhibit the anodic dissolution of the alloy (reaction (7)), which is why **F**_**L**_ decreases j_p1_ (Table 2).

MF always increases the anodic dissolution at a relatively high potential in neutral solutions (Fig. 1, Fig. 3, Fig. 5) for the following reasons.

(1) Fig. 3, Fig. 5 show the decrease in concentration at the interface due to the formation of the surface film without an MF. However, the concentration of the soluble species at the interface increases at high potential under an MF (Fig. 3e1 and 5e1), indicating that the MF accelerates the dissolution rate of Fe and Ni.
(2) As shown in Fig. 9, the content of N is higher without an MF (Fig. 9E1) than that before the test (Fig. 9E) because complex ions form. MF decreases the content of N on the surface because **F**_**L**_ drives the complex ions away from the interface to the solution.

The concentration of the active intermediate (NiSCNH^+^) is lower in the neutral solution than the acidic solution, leading to the active intermediate effects in the neutral solution by **F**_**L**_**.** Table 1, Table 2 show that j_corr_ and E_corr_ are much higher and more negative, respectively, in the acidic solution compared to the neutral solution because insulating oxide films cannot be formed in an acidic medium. However, at the high potential of 1.1 V (Fig. 5, Fig. 6), the anodic current is much higher in the neutral solution than in the acidic solution, which can be explained as follows.

(1) **F**_**L**_ induces convection in the yellow and blue areas in the neutral solution (Fig. 5). However, the concentration increases in the yellow area due to the formation of metal ions, while it decreases in the blue area due to the formation of complex ions. The convection in the blue area is much weaker in the acidic solution (Fig. 6) than in the neutral solution (Fig. 5), indicating that it is difficult to form complex ions in the acidic solution.
(2) The anodic dissolution of the alloy may proceed in steps as shown in Reactions (5), (6), (7) in an acidic solution, in which **F**_**L**_ drives the active intermediate (NiSCNH^+^) away and leaves the surface of the electrode to decrease the anodic current.

According to Canut et al.‘s [5] investigation into the electrochemical properties of nickel-based alloys, the passive layer was predicted to be extremely stable at neutral pH levels (pH 5–6) due to the presence of chromium oxide, nickel, and iron oxide or sulfide based on potential-pH diagrams, providing strong corrosion resistance. However, in the solution containing the aggressive ions (SCN^−^) at the high potential (1.1 V), the oxide film is easily broken, causing pitting corrosion and/or IGC (Fig. 7, Fig. 8). MF always inhibits pitting corrosion by preventing localized acidification [26,27] due to the stirring effect of **F**_**L**_.

Compared to grain bodies, grain boundaries exhibit a more active nature (surface free energy), producing localized IGC (Fig. 5, Fig. 6) by easily absorbing SCN^−^ ions at grain boundaries (Fig. 7, Fig. 8). The Cr-depletion model is the most accepted theory for the occurrence of IGC. According to this model, the lower Cr and higher Fe and Ni contents at grain boundaries [21] promote IGC in SO_4_^2−^ + SCN^−^ solutions (acidic or neutral) under MF. Enhanced anodic dissolution is observed in neutral solution.

## 5 Conclusion

In this work, digital holography (modern) combined with electrochemical methods (conventional) was used to study the anodic dissolution processes of Alloy 690 in SO_4_^2−^ + SCN^−^ solution with and without an MF.

(1) Due to the formation of complex ions ([Ni(NCS)_4_]^2-^ and [Fe(NCS)_4_]^-^), the MF facilitated the anodic dissolution of Alloy 690 in the neutral solution. Due to **F**_**L**_-induced convection, the development of the surface coating was more challenging. However, MF inhibited anodic dissolution in an acidic solution because **F**_**L**_ may drive the active intermediate (NiSCNH^+^) away from the surface of the electrode.
(2) Pitting corrosion and IGC were observed on the surface of the alloy either in acidic or neutral SO_4_^2−^ + SCN^−^ solution. Localized acidification by the stirring effect of **F**_**L**_ was avoided, and pitting corrosion was inhibited in both acidic and neutral solutions.
(3) The contents of Fe and Ni were higher at the grain boundaries than on the grain body. MF promoted the anodic dissolution of Fe and Ni in the form of IGC, whether it increased or decreased the anodic current.

## Author contribution statement

Donglin Xu, Chen Sang: Performed the experiments; Analyzed and interpreted the data.

Boyu Yuan, Liang Li: Conceived and designed the experiments; Contributed reagents, materials, analysis tools or data; Wrote the paper.

## Funding statement

Dr. Liang Li was supported by 10.13039/501100001809National Natural Science Foundation of China [21972059]. Donglin Xu was supported by 10.13039/501100012154Graduate Research and Innovation Projects of Jiangsu Province [KYCX20_2234]. Dr. Boyu Yuan was supported by Tianjin Key Laboratory of Photoelectronic Detection Technology and System [2019LODTS005].

## Data availability statement

Data included in article/supplementary material/referenced in article.

## Declaration of interest's statement

The authors declare no conflict of interest.

## Appendix A Supplementary data

Supplementary material-HELIYON-D-22-22145 Supplementary material-HELIYON-D-22-22145
