# Supplementary material for: Investigation of the effects of the magnetic field on the anodic dissolution of alloy 690 in SO42− + SCN− solution using digital holography
Source: Heliyon. 2023 Feb 9;9(2):e13566. doi: 10.1016/j.heliyon.2023.e13566 (PMC9984449; doi:10.1016/j.heliyon.2023.e13566)
Supplement: Supplementary material-HELIYON-D-22-22145 [file mmc1.docx]

Supplementary material for

Investigation of the effects of the magnetic field on the anodic dissolution of Alloy 690 in SO_4_^2-^ + SCN^-^ solution using digital holography

Dongling Xu^a,#^, Chen Sang^b,#^, Boyu Yuan^b,^*, Liang Li^a,^**

^1^ Jiangsu Key Laboratory of Green Synthetic Chemistry for Functional Materials, School of Chemistry & Materials Science, Jiangsu Normal University, Xuzhou, 221116, China

^2^ Jiangsu Key Laboratory of Advanced Laser Materials and Devices, School of Physics and Electronic Engineering, Jiangsu Normal University, Xuzhou 221116, China

**Correspondence**

Boyu Yuan, Jiangsu Key Laboratory of Advanced Laser Materials and Devices, School of Physics and Electronic Engineering, Jiangsu Normal University, Xuzhou 221116, China. E-mail: [yuanby@jsnu.edu.cn](mailto:yuanby@jsnu.edu.cn)

Liang Li, Jiangsu Key Laboratory of Green Synthetic Chemistry for Functional Materials, School of Chemistry & Materials Science, Jiangsu Normal University, Xuzhou 221116, China. E-mail: [lil@jsnu.edu.cn](mailto:lil@jsnu.edu.cn)

**Funding information**

National Natural Science Foundation of China (No. 21972059) and the Postgraduate Research and Practice Innovation Program of Jiangsu Province (KYCX20_2234) for funding of this project.

**Fig. S1.** H-type electrochemical cell


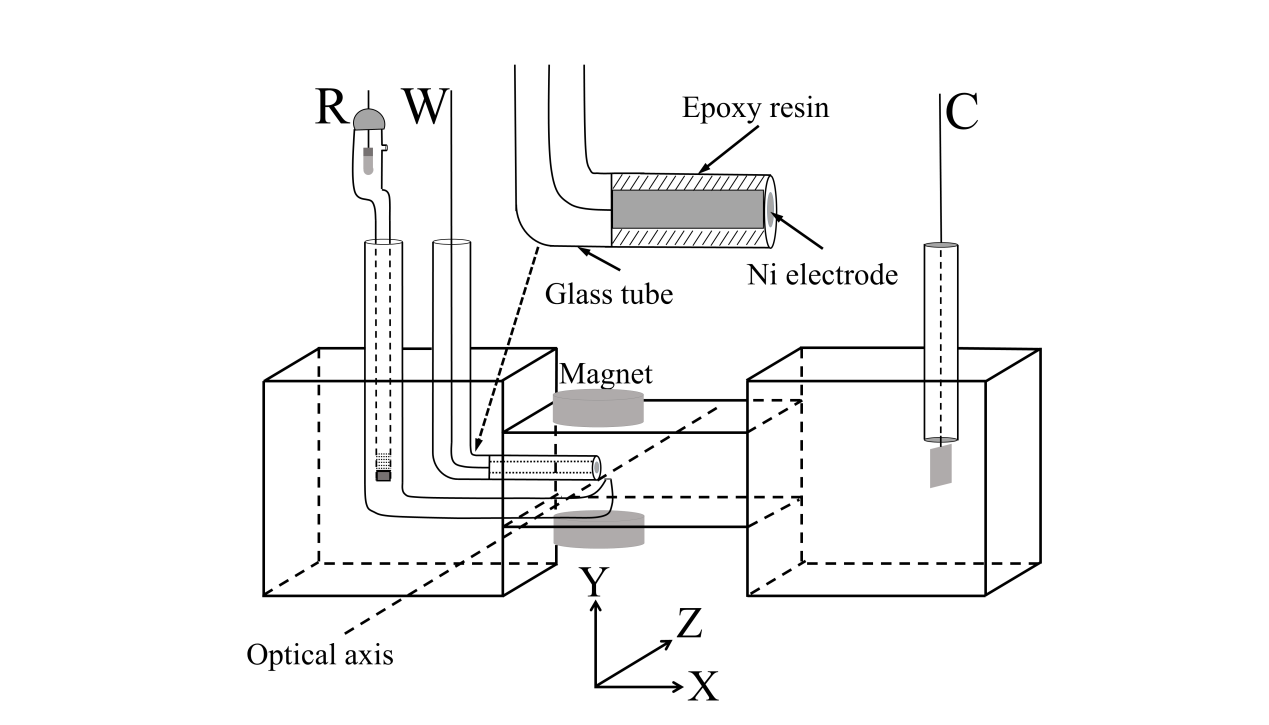


**Fig. S1.** H-type electrochemical cell

W: Inconel 690 electrode; R: saturated calomel electrode (SCE); C: platinum sheet (10 mm × 10 mm); X - horizontal direction; Y - opposite direction of gravity; Z - optical axis direction.

**Fig. S2.** Holographic recording system


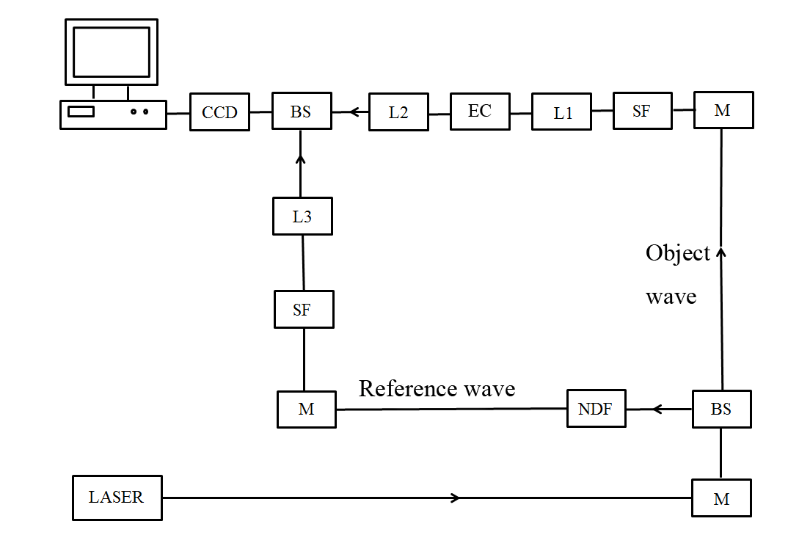


**Fig. S2.** Holographic recording system.

M - mirror, BS - beam splitter, L1 – L3 - collimating lens, SF - spatial filter, EC- electrochemical cell (EB), NDF: neutral density filter.

**Fig. S3.** EDS surface scanning maps in 0.5 M Na_2_SO_4_ + 0.005 KSCN solutions


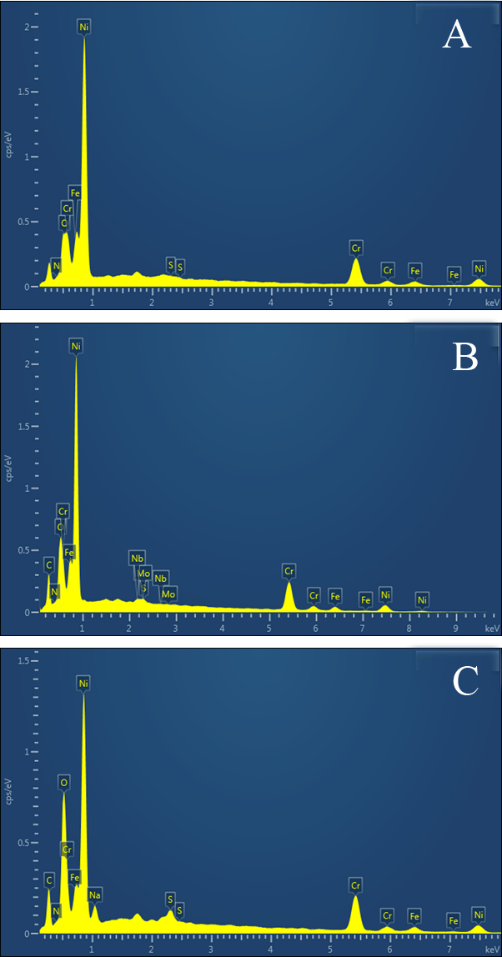


**Fig. S3.** EDS surface scanning maps of the sample corresponding to Figs. 7 A1-C1.

A：Blank；B：After anodic dissolution without MF; C：After anodic dissolution with MF.

**Fig. S4.** EDS surface scanning maps in 0.5 M H_2_SO_4_ + 0.005 KSCN solutions


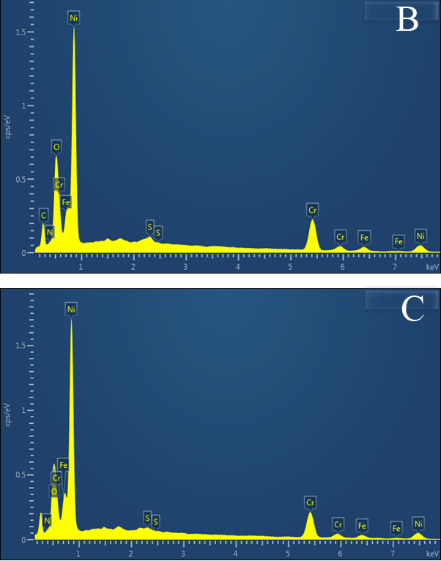


**Fig. S4.** EDS surface scanning maps of the sample corresponding to Figs. 8 B1 and C1.

B：After anodic dissolution without MF; C：After anodic dissolution with MF.
